# Regenerative capacity of the corneal transition zone for endothelial cell therapy

**DOI:** 10.1186/s13287-020-02046-2

**Published:** 2020-12-04

**Authors:** Nicole Ming Sie, Gary Hin-Fai Yam, Yu Qiang Soh, Matthew Lovatt, Deepinder Dhaliwal, Viridiana Kocaba, Jodhbir S. Mehta

**Affiliations:** 1 Tissue Engineering and Cell Therapy Group, Singapore Eye Research Institute, 20 College Road, The Academia, Discovery Tower Level 6, Singapore, 169856 Singapore; 2 Singapore National Eye Centre, Singapore, 168751 Singapore; 3 Department of Ophthalmology, University of Pittsburgh, Pittsburgh, PA 15213 USA; 4 The Netherland Institute for Innovative Ocular Surgery, Rotterdam, The Netherlands; 5 Ophthalmology and Visual Sciences Academic Clinical Program, Duke-NUS Graduate Medical School, Singapore, 169857 Singapore; 6 School of Material Sciences & Engineering, Nanyang Technological University, Singapore, 637551 Singapore

**Keywords:** Cornea endothelium, Corneal endothelial progenitors, Schwalbe’s line, transitional zone, corneal endothelial cell degeneration

## Abstract

The corneal endothelium located on the posterior corneal surface is responsible for regulating stromal hydration. This is contributed by a monolayer of corneal endothelial cells (CECs), which are metabolically active in a continuous fluid-coupled efflux of ions from the corneal stroma into the aqueous humor, preventing stromal over-hydration and preserving the orderly arrangement of stromal collagen fibrils, which is essential for corneal transparency. Mature CECs do not have regenerative capacity and cell loss due to aging and diseases results in irreversible stromal edema and a loss of corneal clarity. The current gold standard of treatment for this worldwide blindness caused by corneal endothelial failure is the corneal transplantation using cadaveric donor corneas. The top indication is Fuchs corneal endothelial dystrophy/degeneration, which represents 39% of all corneal transplants performed. However, the global shortage of transplantable donor corneas has restricted the treatment outcomes, hence instigating a need to research for alternative therapies. One such avenue is the CEC regeneration from endothelial progenitors, which have been identified in the peripheral endothelium and the adjacent transition zone. This review examines the evidence supporting the existence of endothelial progenitors in the posterior limbus and summarizes the existing knowledge on the microanatomy of the transitional zone. We give an overview of the isolation and ex vivo propagation of human endothelial progenitors in the transition zone, and their growth and differentiation capacity to the corneal endothelium. Transplanting these bioengineered constructs into in vivo models of corneal endothelial degeneration will prove the efficacy and viability, and the long-term maintenance of functional endothelium. This will develop a novel regenerative therapy for the management of corneal endothelial diseases.

The cornea is the transparent anterior part of the eye and is composed of five different layers. The outermost layer is the corneal epithelium, followed by Bowman’s membrane, corneal stroma, Descemet’s membrane (DM), and the innermost corneal endothelium. The adult human cornea is about 550 μm thick and serves 3 major functions: (1) as a mechanical and chemical barrier to protect the inner ocular tissues, (2) as a transparent medium for light passage, and (3) light refraction (providing two thirds of the eye’s refractive power) [1]. Inside the corneal stroma, the orderly arrangement and interfibrillar spacing of collagen fibrils regulate the light transmission through the cornea [2]. Increased light scattering occurs when the spatial arrangement, diameter, and densities of collagen fibrils are altered and when an alteration in the refractive index ratio between fibrils and the extrafibrillar matrix is encountered [3]. All these factors are influenced by the stromal hydration status, as fluid sequestration within the stroma is expected to separate the constituent collagen fibrils, altering the fibrillar spacing and alignment. On the posterior corneal surface, a single layer of corneal endothelial cells (CECs) are arranged in a tightly packed tessellated pattern on its basement membrane, the DM, which forms a barrier between the corneal stroma and aqueous chamber [4, 5]. CECs are metabolically active and facilitate a continuous pumping action with a fluid-coupled active efflux of ions from the corneal stroma into the aqueous humor [6]. The efficient pumping activity prevents stromal over-hydration and maintains an orderly arrangement of collagen fibrils with precise spacing for undisturbed light passage, which is essential for corneal transparency.

In vivo, postnatal human CECs are terminally differentiated, non-mitotic cells, which are arrested in the G1 phase of the cell cycle [7]. A multitude of factors, including cell-cell contact inhibition, the presence of negative cell cycle regulators (including cyclin-dependent kinase inhibitors, p27^kip1^ and p15^INK4b^), growth inhibitors (like transforming growth factor-β, TGFβ) present in the aqueous humor, and stress-induced premature senescence, contribute towards the maintenance of CECs in their non-regenerative state [8, 9]. Mean CEC density is the highest in neonates (at 3000–4000 cells/mm^2^) and declines slowly at an estimated rate of about 0.6% per annum, falling to ~ 2500 cells/mm^2^ in early adulthood and ~ 2000 cells/mm^2^ in old age [10, 11]. The cell loss is further accelerated by degenerative diseases, such as in Fuchs endothelial corneal dystrophy (FECD), infections (e.g., viral endotheliitis), inflammation, or intraocular surgical trauma [12]. Since CECs are non-replicative, the normal response to CEC attrition involves neighboring healthy CECs to undergo lateral migration and expansion, resulting in clinically observable characteristics of polymegathism (CEC enlargement) and pleomorphism (loss of CEC hexagonality) [13]. CEC density of approximately 500 cells/mm^2^ represents the minimum cell density required to sustain the corneal endothelial pumping efficiency. Further CEC attrition beyond this threshold results in an overall endothelial pump failure (endothelial decompensation), characterized clinically as corneal edema, with the attendant manifestations of visual disturbance and eventually corneal blindness. Loss of stromal deturgescence may be accompanied by frank corneal epithelial edema and the formation of bullous epithelial cysts, the rupture of which results in severe pain, in a condition known as bullous keratopathy [14].

The current standard of care for patients with advanced corneal endothelial degeneration is allogeneic corneal transplantation, or keratoplasty, which surgically replaces the affected patient’s cornea with a healthy cadaveric donor cornea, to restore corneal clarity. Although penetrating (full-thickness) keratoplasty (PK) was previously the most commonly performed procedure, innovations in surgical techniques have led to the development of endothelial keratoplasty (EK) techniques, including Descemet’s stripping automated endothelial keratoplasty (DSAEK) and Descemet’s membrane endothelial keratoplasty (DMEK) [15, 16]. The major advantages of EK over conventional PK are that it significantly reduces the risk of postoperative graft rejection and avoids the use of long-term steroids, as well as the achievement of rapid postoperative visual rehabilitation and preservation of tectonic stability [17]. However, despite the excellent outcomes of EK, the worldwide supply of cadaveric donor tissue is still inadequate to address the critical need for corneal transplantation [18, 19]. The development of alternative therapeutic modalities to treat corneal endothelial diseases, either independent of or in a way that is less reliant on the supply of donor tissues, represents an urgent clinical unmet need. In recent years, this is a subject with intense research interest worldwide [16, 20–22].

Several strategies have been under investigation with experimental and early clinical successes. They include cell therapy, whereby CECs from donor corneas are cultured and expanded in vitro, followed by the administration into the anterior chamber, either as a cell suspension or as a cell sheet [23–27], and the augmentation of CEC proliferation with Rho-associated kinase (ROCK) inhibitor to promote corneal endothelial healing [28, 29]. A recent landmark study by Kinoshita et al. demonstrated that the intracameral injection of donor CECs in the presence of ROCK inhibitor was able to reverse corneal edema with stable clinical outcomes on patients with bullous keratopathy and FECD [30]. The major advantage of cell therapy is that the ex vivo cultivation of cells from a single donor cornea may potentially be expanded to treat many patients. However, the practical difficulties of developing good manufacturing practices (GMP)-compliant cell manufacturing process in a xeno-free setting, to produce consistently high-quality cells, while preserving the desired morphological and functional characteristics, may restrict the application in many countries [15, 16].

To circumvent the technical, regulatory, and logistical issues related to ex vivo cell culture followed by the expansion of an allogenic population of donor CECs, one may consider the possibility of treating corneal endothelial disease via the stimulation of endogenous CEC regeneration. While various modes of endogenous regenerative therapy, such as the acellular Descemet’s membrane transplantation (DMT), Descemet stripping only (DSO), and selective endothelial removal (SER) have recently been described, the CEC regeneration induced by these surgical procedures appear to be related to the lateral migration and enlargement of healthy CECs from the unaffected periphery [21, 31–33]. True endogenous CEC regeneration, however, is defined by the inducement of CEC mitosis, via the provision of external stimuli to unlock CECs from their quiescent state of G1 arrest in the cell cycle. In recent years, evidence has been accumulating to suggest the presence of a putative population of endothelial progenitors in the peripheral endothelium (PE). Unlike central CECs, these progenitors harbor stem cell-like characteristics, with the implication that they may be more amenable to proliferation-stimulation strategies. This has opened a new avenue of research, targeted towards the identification, in vitro propagation, and differentiation of immature CECs, as a novel therapeutic modality for the management of corneal endothelial diseases.

## Embryonic development of corneal tissues

Eye development begins in the third week through to the 10th week of gestational age, and corneal tissue formation is initiated at about the 5th to 6th week of gestation [34]. The cranial neural crest derives 3 distinct layers, namely epidermal ectoderm, neuroectoderm, and periocular mesenchyme (POM), that make up the embryonic origins of the mammalian eye. In vertebrates, neural crest cells (NCCs), a population of multipotent stem cells originate from the neural plate, migrate to different regions of the embryo to form a broad range of tissues (including endocrine cells, melanocytes, peripheral neurons, cartilage, and bones) [35]. At the cephalic level, NCCs form the mesectoderm, which gives rise to craniofacial connective, dermal and skeletal tissues, and cranial ganglia [36]. In the eye, NCCs from prosencephalon (developing forebrain) and mesencephalon (developing midbrain) migrate to the periocular region to form POM, which differentiates into various structures of the anterior segment [34]. POM cells migrate into the space between the lens vesicle and surface ectoderm, which is the first wave of NCC migration, forming the corneal endothelium [12, 35]. The second wave of NCC migrates into the space between the immature epithelium and endothelium, giving rise to primitive keratocytes, which, together with corneal epithelium, synthesize the primary stroma matrix components [37]. The third wave migrates towards the angle between the posterior cornea and the anterior edge of the optic cup, contributing to iris stroma and ciliary body. The POM located anteriorly to this angle initially remains undifferentiated but then develops into cells comprising trabecular meshwork (TM) and Schlemm’s canal, respectively [12, 35, 37].

Given that NCC and POM differentiate into various ocular tissues, the exact mechanisms underlying these processes are not fully understood. A combination of signaling molecules, such as TGFβ and Wnt [38–40], and transcription factors, such as Pax6, Foxc1, Foxc2, and Pitx2, have been reported to play different regulatory roles [34, 40, 41]. Further studies examining these molecular mechanisms are still necessary to better understand the factors involved in the terminal differentiation of POM into different cell lineages. In adult corneas, POM cells have been found to exist as cells co-expressing HNK1 and p75 neurotrophin receptor (p75^NTR^) (NCC markers) and Pitx2 (POM marker) in a region between PE and TM, referred to as the transition zone [41].

## Corneal endothelial periphery and transition zone

At various locations of the corneal endothelium, CECs appear with different features. In humans, CECs are present at higher densities in the paracentral and peripheral regions than centrally [42]. Ex vivo studies using nuclear staining performed on fixed and fresh specimens found that the CEC count was 3632 ± 592 per square millimeter in PE and 2778 ± 284 per square millimeter in central corneal endothelium (CE). A similar trend was also found by staining with Alizarin-red [43]. In vitro studies on cell growth features also revealed that cells in the PE had a higher mitogenic activity with less tight cell-cell adhesion than cells from CE [44]. The sphere-forming propensity by PE cells further demonstrated their proliferative potential [45, 46]. Clinically, transplantation studies have supported the migration of recipient PE cells to replace or co-exist with donor endothelial cells, after centrally implanted PK [47, 48]. All these observations suggest the PE has a greater proliferative response than the CE.

Evidence has suggested that the endothelial periphery contains less differentiated cells and representative studies are summarized in Table 1. At the most peripheral region, cells expressing telomerase and replicating DNA, detected by BrdU incorporation, have been identified [49]. The authors proposed that these cells could represent the progenitors of corneal endothelial and TM cells, and cell migration might occur following injury and could be age-dependent. McGowan et al. identified cells expressing stemness markers, which were nestin, alkaline phosphatase, and telomerase positive, present in the transition region between the PE and TM. Also, Oct3/4, Pax6, Sox2, and Wnt-1 were demonstrated to be expressed after corneal endothelial injury [50]. A subpopulation of slow-cycling, label-retaining cells expressing nestin and nerve growth factor receptor were also found to reside at the endothelial periphery in corneas from adult mice [57]. These findings thus demonstrate the presence of stem-like progenitors in the region spanning from the endothelial periphery to TM. Recently, Yun et al. identified TM stem cells at the insert region under the Schwalbe’s line [58]. This region does not filter aqueous humor into the Schlemm’s canal but contains cells expressing stem cell and neural crest markers (ABCG2, myc, nestin, p75^NTR^, N-cadherin). These cells have been demonstrated to undergo multipotent differentiation into corneal endothelial cells, adipocytes, and chondrocytes, but not osteocytes or keratocytes [59, 60] (Table 1). However, no data has been provided on whether the TM-derived endothelial-like cells could display specific physiological activities, i.e., pumping function and trans-endothelial resistance.

**Table 1:** Summary of studies reporting human corneal endothelial progenitors with potential location, identification, and differentiated functions

Possible location of endothelial progenitors	Methods of identification	Markers	Remarks	References
Not specific in corneal endothelium	Sphere-forming assay	Nestin, GFAP, β3-tubulin, αSMA	Dissociated sphere cells showed hexagonal shape and pumping activity; no p75^NTR^ expression.	[45]
Peripheral endothelium	BrdU labeling and immunostaining	Alkaline phosphatase, telomerase	Progenitors in a niche at the junction between corneal endothelium and TM.	[49]
TM and transition zone between TM and corneal periphery	Corneal wounding model and immunostaining	Alkaline phosphatase, nestin, telomerase, Oct3/4, Pax6, Wnt1, Sox2	Wounding activated Oct3/4 and Wnt1 expression as a response to initiate the endothelial repair process.	[50]
Peripheral endothelium	Sphere-forming assay	Nil	PE had a significantly higher percentage of sphere formation, representing precursor density.	[51]
Peripheral endothelium	Immunostaining and flow cytometry	Lgr5, Hedgehog pathway markers (SHH, Gli1, Gli2)	Lgr5+ cells were proliferative. Generation of differentiated corneal endothelium and functional assay was not demonstrated.	[52]
Central and peripheral endothelium; progenitor enriched at transition region between CE and TM	Immunostaining and flow cytometry	P75^NTR^, Sox9, FoxC2	Expressed partial properties of neural crest and periocular mesenchyme; differentiated cell sheet had pumping activity by Ussing chamber system and in vivo transplantation to rabbit corneas.	[53]
Whole corneal endothelium of normal and FECD corneas	Colony-forming populations; > 80 passages	Pax3, nestin, Sox9, AP-2β, p75^NTR^, Sox2, Lgr5, p63, Oct4	Adult corneal endothelium harbored neural crest-derived progenitors capable of perpetual proliferation and formation of endothelial layer exhibiting trans-endothelial resistance.	[54]
Trabecular meshwork	3D Matrigel culture to activate BMP signaling	AQP1, MGP, CHI3L1, AnkG, Oct4, Sox2, Nanog, ABCG2, p75^NTR^, FOXD3, Sox9, Sox10, MSX1	TM progenitors were multipotent to differentiate into corneal endothelial cells, adipocytes, and chondrocytes.	[55]
Transition zone (inner TZ)	Immunostaining, cell culture	Lgr5, telomerase, nestin, Sox2, p75^NTR^, Pitx2, HNK1	Inner TZ, adjacent to PE, contained progenitors that projected as multicellular clusters into PE. Porcine TZ progenitors differentiated to endothelial monolayer expressing ZO1 and Na^+^K^+^ATPase.	[56]

Using gonioscopy or optical coherent tomography (OCT), this region at the posterior limbus contains the most peripheral endothelium, Schwalbe’s line (marking the termination of Descemet’s membrane), and the anterior non-filtering portion of TM [61, 62]. In a rhesus monkey model, cells present in Schwalbe’s line showed unique features different from CECs and TM cells, such as the formation of discontinuous cord with circumferential orientation and the presence of prominent Golgi apparatus and cytoplasmic inclusions [63]. In a canine glaucomatous eye model, the number and secretory appearance of these cells were found to change with disease progression, suggesting an association with the non-filtering portion of TM [64]. The proliferative capacity of these Schwalbe’s line cells has also been documented in studies of argon laser trabeculoplasty for glaucoma treatment. Using thymidine incorporation assay, TM cell repopulation after surgery was related to increased cell division [65]. More than 60% of the dividing cells were localized to the anterior non-filtering region of TM, suggesting the cell proliferation and migration from near Schwalbe’s line [66]. In some cases, the cell overgrowth even blocked the aqueous humor outflow, leading to the failure of the surgery. These observations lead to the speculation that Schwalbe’s line cells might have a proliferative capacity.

However, using scanning electron microscopy (SEM), a thin annulus of smooth or flat tissue, adjacent to DM terminus, termed the transition zone (TZ), has been revealed. Interestingly, the TZ is devoid of CEC and TM fibers [67, 68]. This region is demarcated with the inner border adjacent to the PE as shown by a transition from regularly arranged tightly packed PE cells to more polygonal cells with irregular sizes inside the TZ [56]. The outer border is marked by the anterior non-filtering portion of TM with beam inserts and bridges. Several studies using SEM had reported the average width of human TZ ranges from 80 to ~ 200 μm, using random, block area to 360° whole rim measurements [56, 68, 69]. As most of these studies were conducted using Caucasian donor corneas from middle to old age (with the mean CEC density > 2000 cells/mm^2^), it is as yet not known if there is any association of TZ dimension with donor age and corneal health. Our recent analysis comparing two age groups, ≤ 60 years old (*n* = 6) versus > 60 years old (*n* = 11), showed no statistically significant age-related changes; however, the sample size was small [56]. Hence, we cannot exclude any TZ variation, especially in young donors. Further studies involving a larger sample size, from different ethnicities, and also corneas from degenerative diseases, will give us more information on the TZ status in corneal health.

**Fig. 1:**
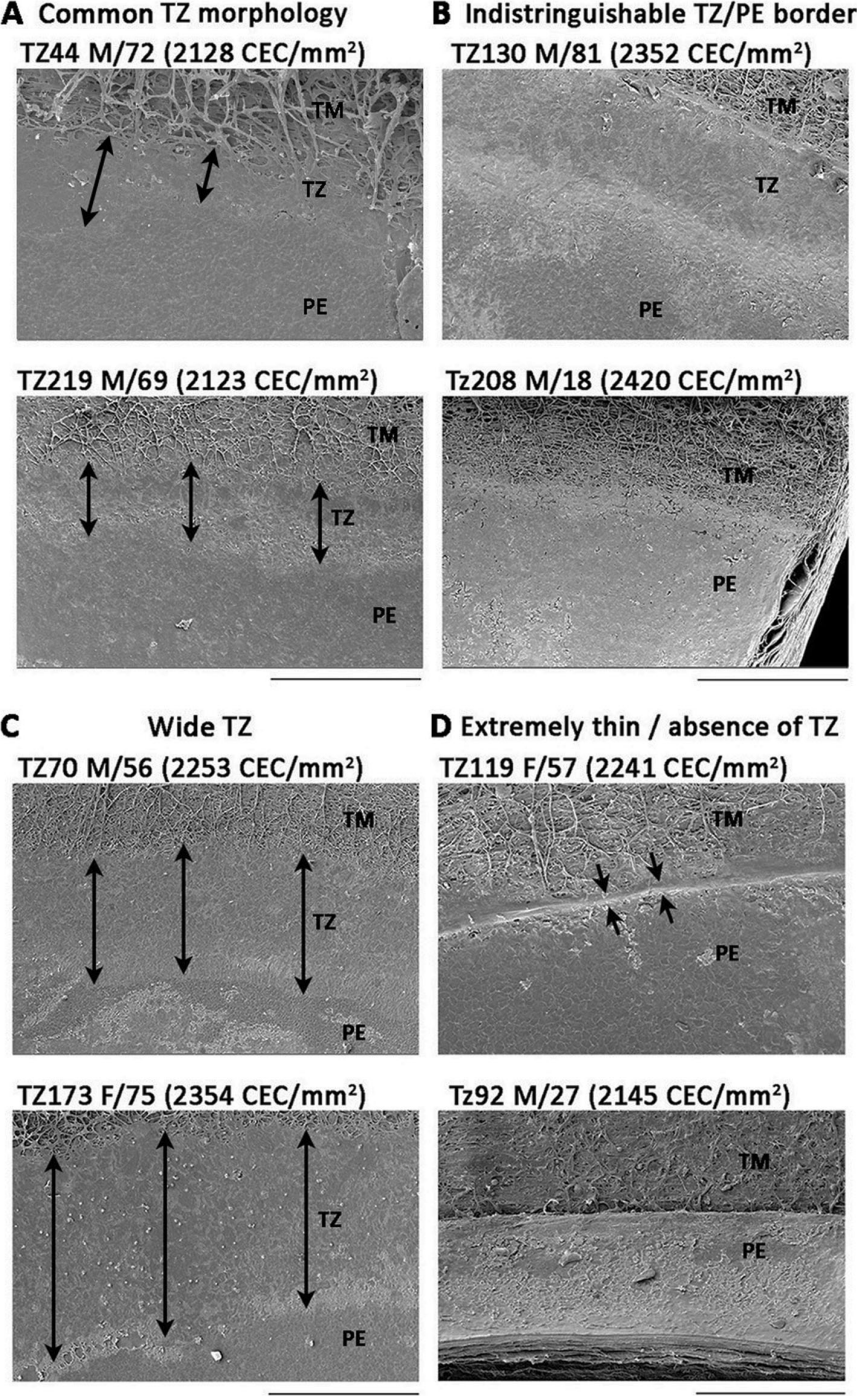
Human transition zone variation. **a** Typical TZ morphology with distinguishable smooth TZ (double-head arrows) between PE with cobblestone pattern of endothelial cells and TM with trabeculae inserts and bridges. **b** Indistinguishable TZ with an unclear border of PE. **c** Wide TZ with average width > 500 μm. **d** Absence of TZ, with a deep cleft located between TM and PE. CEC, corneal endothelial cells; PE, peripheral endothelium; TM, trabecular meshwork; TZ, transition zone; M, male; F, female. Scale bars, 300 μm

The TZ width distribution has been shown to vary in different anatomical locations. In orientation-marked corneas, the nasal TZ width was found to be significantly thinner (a ratio of 0.8 compared to the TZ in the superior quadrant, and 0.9 in the inferior and temporal TZ) [56]. Similar findings were also reported by Breazzano et al. [69]. Nasal polarization has been commonly observed in mammalian eyes. There is a greater distribution of blood and lymphatic vessels in the nasal limbus of normal corneas [70, 71]. Pterygium also predominantly affects the nasal side of human corneas [72]. Besides, TZ with an atypical appearance has been observed, even in donor corneas with high CEC densities (> 2000 cells/mm^2^). Compared to corneas with typical TZ, some samples had an indistinguishable border between TM and PE, hence imposing difficulty in identifying the TZ. There were also samples with much wider TZ (> 700 μm) and samples with extremely thin or even absence of TZ resulting in the PE located near TM (Fig. 1). Samples showing a deep cleft at the TZ location were also observed. Hence, variable TZ features could occur in corneas with good central CEC count. Whether TZ variations affect corneal health or cause any physiological consequences is yet to be investigated.

**Fig. 2:**
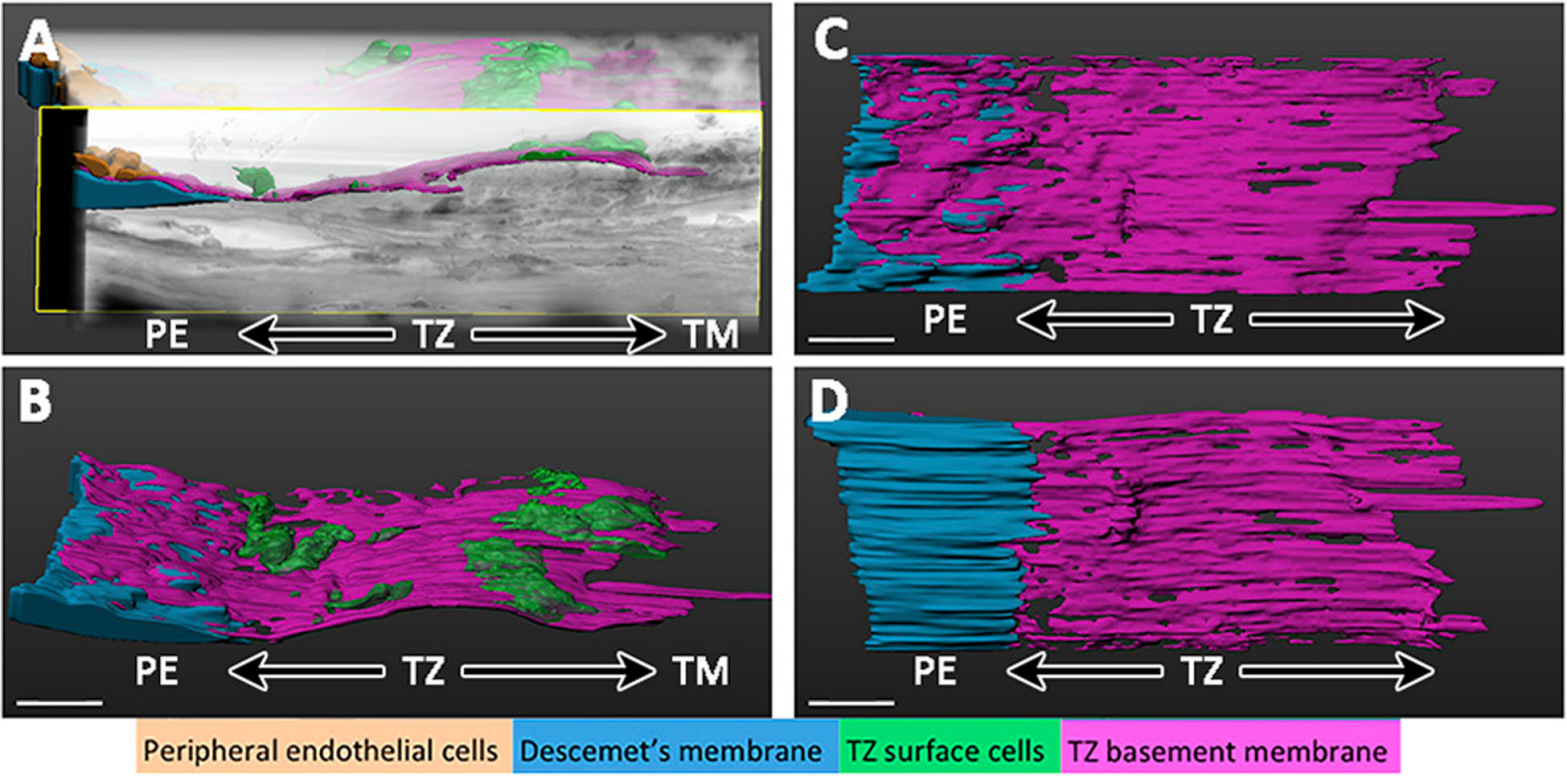
Serial block face-scanning electron microscopy of the junction between TZ and PE and 3D reconstruction. **a** Batch-aligned pack of transmission electron microscopy (TEM) slices showing an overview of PE/TZ junction. **b** 3D reconstructed image of TZ/PE junction showing DM insertion below the TZ surface. **c** En face view showing the TZ surface overlaying the DM. **d** Posterior view showing the insertion of DM beneath TZ. Blue, DM; purple, TZ surface; green, TZ surface cells; brown, endothelial cells; DM, Descemet’s membrane; PE, peripheral endothelium; TM, trabecular meshwork; TZ, transition zone. Scale bars, 30 μm

The TZ border with PE has been recently examined using serial block face-scanning electron microscopy and three-dimensional (3D) reconstruction. The very terminus of DM was found to taper and insert beneath the TZ surface and this extended as far as 1/3 to 1/2 of the entire TZ breadth, indicating a potential bridging zone between these 2 regions (Fig. 2) [56]. Whole-mount confocal immunofluorescence and immuno-SEM further distinguished 2 phenotypically distinct regions inside the TZ, in which the inner TZ, i.e., adjacent to PE, contained cells expressing various markers associated with stemness (ABCG2, CD34, Lgr5, and telomerase), and cells in the outer TZ connecting to TM, had cells expressing fibrosis markers (positive to CD90 and vimentin). Under transmission EM (TEM), in the stromal region of the inner TZ, stem-like cells with a large nuclear-to-cytoplasmic ratio were detected, while the outer TZ had more pigmented cells. These observations indicated that the inner TZ can be a progenitor-enriched region. We identified the presence of finger-like extensions of multicellular clusters containing Lgr5 positive cells from the TZ into the PE, also supporting the concept of progenitor migration [56]. Lgr5 (leucine-rich repeat-containing G protein-coupled receptor 5) is uniquely expressed in PE cells and could have a homeostatic role in maintaining endothelial phenotypes and inhibiting mesenchymal transition through Hedgehog and Wnt regulation [52]. Whether the cell movement from the TZ to PE has any association with the DM terminus that inserted into the TZ stroma, or if the DM terminus acts as a biomechanical guide to direct TZ cells into PE, has to be investigated.

The multicellular clustering continues into the extreme PE region (approximately 200 μm of the endothelium). He et al. described that PE cells were organized in small clusters with 2–3 cell layers around the Hassal-Henle bodies [73]. This arrangement was associated with linear rows of cells that centripetally aligned in the PE. Under SEM, DM furrows were detected and the endothelial cell nuclei were lodged at the base. Compared to the central endothelium, PE cells expressed less endothelial markers, including ZO-1, Na^+^/K^+^-ATPase, and COX IV, but preferentially expressed stemness-related nestin, telomerase, and ABCG2. Based on this anatomical organization that was mainly documented in old donor corneas (age of 80 ± 12 years), the authors theorized a slow centripetal migration of less differentiated cells in the endothelial periphery, throughout life.

## Transition zone in different animal species

**Fig. 3:**
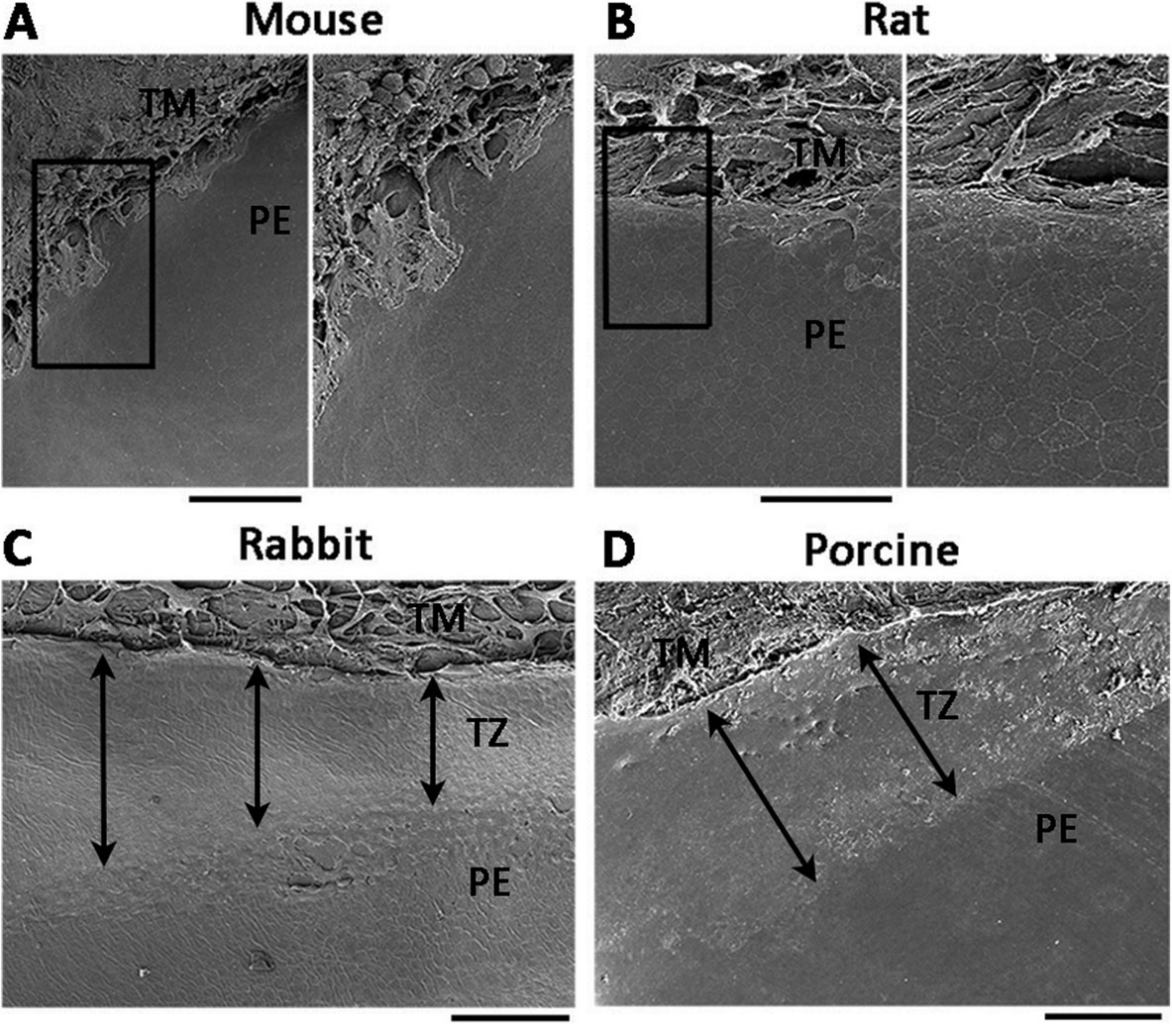
TZ in different animal species. **a** Mouse and **b** rat with indistinguishable TZ. Insets showing a lack of TZ structure. Insets and magnified images. **c** Rabbit and **d** porcine TZ with a clear smooth zone between PE and TM (double-head arrows). PE, peripheral endothelium; TM, trabecular meshwork; TZ, transition zone. Scale bars, 300 μm

Reports on the appearance of TZ in the posterior limbus of non-human species are lacking. Corresponding to the human TZ and its positioning with the adjacent PE and TM, bovine samples showed a very different anatomical organization that the transition between PE and TM being very abrupt with no apparent TZ observed [68]. The lack of distinct TZ structure was similarly found in murine corneas (adult mouse and rat) (Fig. 3a, b). The anterior trabeculae of TM architecture were transited immediately to the extreme PE, which displayed regularly arranged hexagonal cells. The smooth TZ was negligibly detected. In contrast, adult rabbit and porcine corneas showed well-demarcated TZ, located between PE and TM, similar to that in humans (Fig. 3c, d). The TZ surface was covered by flattened polygonal cells, which became continuous with the tightly packed PE cells. The posterior edge was distinct from the border outlined by the TM trabeculae. The evidence of stem cells in animal TZ has also rarely been reported. In bovine corneas, while the entire corneal endothelium, TM, and TZ were positively labeled with different stem cell markers, including ABCG2, nestin, Oct4, Pax6, Sox2, STRO-1, and telomerase, the TZ exclusively expressed ankyrin G, a marker of TM insert cells [68]. Besides this report, no similar work examining TZ of other animal species has been performed, probably due to the lack of availability of suitable antibodies across species. Hence, there exists appreciable interspecies variation in the TZ existence and structures. Whether this structural divergence relates to the different localization of putative endothelial progenitors and their proliferative capacity has to be examined. Espana et al. reported that slow-cycling cells expressing progenitor markers existed in the periphery of mature corneal endothelium in mice [57], though an identifiable TZ did not exist in the mouse corneas.

## Ex vivo propagation of TZ cells and generation of the corneal endothelium

To establish TZ stem cell culture and avoid the possibility of suboptimal viability of human TZ cells from cryopreserved donor corneas, fresh porcine ocular tissue within approximately 6 h of death would be a feasible cell source. Investigators have attempted to improve the cell isolation and culture conditions to maximally amplify cells while retaining the cell morphology and marker expression after differentiation. Porcine TZ has been isolated free of PE and TM tissues and digested with collagenase to obtain single cells for culture [56]. In the proliferative phase, the primary cells expressed the POM marker Pitx2, indicating their undifferentiated status. At confluence, they were able to generate a homogenous monolayer of endothelial-like cells with characteristic cell surface expression of pump-associated Na^+^/K^+^-ATPase and junctional complex ZO-1, while the Pix2 expression was downregulated. Whether such ex vivo proliferative and regenerative capacity can be demonstrated with human TZ cells is under investigation. Recently, Zhang et al. reported endothelial cell regeneration from human “wider TZ” (~ 2 mm width), comprising of Schwalbe’s line, a portion of PE and TM, by explant culture. They showed the proliferation of endothelial-like cells with the expression of ZO-1, Na^+^/K^+^-ATPase, and Col8A2 [55]. However, the source of cultured cells from TZ or PE was not determined. Isolation of a single cell type from TZ or PE should be attempted to obtain a homogenous cell population that can facilitate future research on corneal endothelial cell differentiation, the study of mechanisms of action, and potential translational application as an endothelial regenerative therapy.

## Cell culture of endothelial progenitors

In vitro, human corneal endothelial progenitors (CEPs) have the propensity to form spheres, which are the clonal colonies generated in culture on low adhesion surfaces, and demonstrate the cellular capability to proliferate [45]. This feature of colony-forming could be related to the cell origin from NCC. Cells inside spheres are small and have BrdU retention ability, suggesting their progenitor nature. The colonies adhered and differentiated to CEC-like with a hexagonal shape and demonstrated fluid transference with an Ussing chamber assay. When the spheres were injected into the anterior chamber of rabbit eyes with corneal endothelial deficiency, CEC-like cells were detected on the recipient’s DM with restored endothelial functions, and this resulted in reduced corneal edema [74]. Also, human CEPs generated a single cell layer on the denuded human amniotic membrane and exhibited typical hexagonal shape and tight junctions (ZO-1 positive) [75]. These spheres also displayed different features for the culture conditions. Noh et al. showed different cell phenotypes, including the ability to express corneal endothelial markers (ZO-1, Col8A2, Na^+^/K^+^-ATPase) or undifferentiated markers (e.g., Oct3/4, nestin) and the ability to undergo endothelial-mesenchymal transition, when the spheres were cultured in different media [76]. In serum-free culture, human CEPs retained partial features of NCC and POM and highly expressed NCC markers (including p75^NTR^, SOX9, and FOXC2) [77]. Besides, CEPs proliferated more efficiently on a surface coated with laminin-511, than other substrates, and displayed bipolar, spindle-shaped morphology, was similar to that of NCCs [78].

The ability of human CEPs to proliferate in culture varies among different studies. Hara et al. observed a higher proliferative capacity in young donors (< 60 years) as compared to corneas from the elderly (> 60 years). A maximum of 5 passages was recorded, with a decline seen in p75^NTR^ expression with each recorded passage [77]. Yokoo et al. reported CEP sphere formation was maintained for the first 2 passages and, after re-plating, the efficiency decreased dramatically between the primary sphere colonies and secondary colonies [45]. PE was also demonstrated to contain a higher density of CEPs deriving spheres than that from CE [51]. In contrast, Katikireddy et al. identified a rapidly proliferating population of cells from human corneal endothelium that exhibited NCC-derived progenitor features, such as sphere-forming capability, absence of senescence, and high colony-forming efficacy during sub-passaging [54]. The cells could be maintained beyond 80 passages and grew continuously in regularly shaped hexagonal monolayers with contact inhibition. The endothelial-mesenchymal transition was not detected as fibroblast-like cells underwent senescence within passages. This could be related to the blockage of the canonical Wnt-Smad2/3 pathway during the ex vivo culture. However, which factors were responsible for inhibiting these signaling and maintaining the progenitor phenotypes have yet to be further investigated. In our preliminary data, human TZ cells could propagate from < 100 viable cells from fresh isolation to > 500,000 cells after 5 passages and the cell progeny maintained the differentiation capability of CE progenitors on acellular DM (data not shown).

## Other experimental sources of corneal endothelial progenitors

Human embryonic stem cell (ESC) research has offered great potential in cell differentiation, with theoretically an unlimited supply of specific cell types. Such ability to pre-determine cell fate and produce multiple cell lineages holds vast possibilities in the field of regenerative medicine. This has sparked a revolution in the management of several diseases, such as Parkinson’s disease and diabetes [79–81]. In the cornea, a few studies have reported the differentiation of ESCs to express CEC-like phenotypes. Zhang et al. showed the in vitro differentiation of human ESCs to periocular mesenchymal precursors (POMP), which then generated CEC-like cells [82]. The differentiated cells expressed N-cadherin, transcription factors FoxC1 and Pitx2, indicating CEC differentiation. Culturing these cells on acellular porcine stromal matrix lamellae produced a monolayer endothelial-like cell sheet that showed functionality when used as a tissue-engineered construct in a rabbit model of bullous keratoplasty. In another study, mouse ESCs were induced to undergo NCC differentiation by retinoic acid, and subsequent differentiation in lens epithelial cell-conditioned medium generated cells expressing CEC phenotypes (ZO-1, NA^+^/K^+^-ATPase, N-cadherin, aquaporin-1) [83]. Alternatively, ESCs were first induced to NCC by the treatment of TGFβ inhibitor (SB431542) and Noggin (“dual Smad inhibition”), then subsequent blocking of Wnt/β-catenin signaling by DKK-2, in the presence of cytokines (basic fibroblast growth factor and platelet-derived growth factor B) generated cells showing hexagonal shape and expression of ZO-1, NA^+^/K^+^-ATPase, and Col8A1, resembling CECs [84].

Induced pluripotent stem cells (iPSCs) contain the essential properties of ESCs. Since their first report in 2006, the reprogramming techniques have rapidly evolved and iPSCs have become a powerful “de-differentiated” cell type that can be deployed to generate specific cell lineages [85]. In corneal endothelial studies, iPSCs were reported to differentiate into CEC-like cells using a modified McCabe’s protocol as described above [86]. Likewise, the chemically defined conditions using small molecules for “dual Smad inhibition” together with the modulations of BMP and Wnt pathways also generated cells displaying CEC-like features from iPSCs via NCC induction [87]. Due to more ethical acceptance, research of tissue-specific adult stem cells or progenitors is being investigated. However, in contrast to ESCs and iPSCs, they have limited differentiation capacity.

A prominent role of the corneal endothelium is its barrier function. The expression and localization of barrier-associated proteins, such as cadherin protein CDH2, and other adhesion-associated proteins (CLDN11, TMEM204, and TMEM178A) and glycocalyx-associated proteins (APOE, MYOC, DCN, LUM, and APOD) have revealed the organization of adhesive interactions between cells [27]. The expression of tight junction ZO-1 protein, encoded by TJP1 gene, is an important scaffolding component, regulating the paracellular diffusion of ions and solutes, and maintaining the endothelial cell polarity [88]. Freeze-fracture electron microscopy of transmembrane fibrils and immunostaining for ZO-1 and occludin in tight junction complex has provided a qualitative insight into the barrier integrity [89]. Moreover, the high expression of transporter proteins (SLC4A4, SLC4A11, and SLC16A1) has been shown to maintain the solute gradients across the plasma membrane to regulate fluid transport. In vitro function of these transporters has been measured by the intracellular proton concentrations (pH_i_) over time. In native CECs, these channels can establish a membrane potential of about − 30 mV [90]. Mutations in SLC4A11 have been associated with endothelial diseases, e.g., FECD and congenital hereditary endothelial dystrophy [91–93].

The integrity of tight junction dynamics in the endothelial monolayer has been examined using an Ussing chamber system, by which the trans-endothelial electric resistance (TEER), measures the ohmic resistance or impedance across a spectrum of frequencies. This reflects the ionic conductance of the paracellular pathway in the cell monolayer [94]. Studying the flux of non-electrolyte tracers in this system can also indicate the paracellular water flow, as well as the pore size of tight junctions [95]. The microfluidic implementation on TEER assay (Organs-on-chip system) has allowed the study of the physiological effects of parameters, such as fluid flow conditions and shear stress, that could induce a mechano-transductive effect on the cell monolayer [96]. The small culture volume and the immobilized TEER electrodes placed close to the cell monolayer can reduce the electric resistance and signal noise from the culture medium, and this can ensure a uniform current density for non-erratic TEER values [97]. Organs-on-chips may also allow the incubation of endothelial cell monolayer in aqueous humor to achieve physiological relevant situations.

Animal models (e.g., rabbit or non-human primate) are desirable to prove the physiological endothelial functions and safety. Transplantation of a monolayer of endothelial cell sheet (akin to a DSAEK/DMEK procedure) or cell injection in animal models of corneal endothelial damage, such as after central descemetorhexis, followed by ocular evaluation for corneal clarity and edema (by measurement of corneal thickness), has been demonstrated to restore endothelial function in regulating stromal hydration [26, 77].

## Perspective and challenges

The identification and propagation of CEPs from PE and TZ of donor corneas or autologous corneal tissues could represent a novel cell source to generate corneal endothelium for regenerative medicine. There is certainly an ample availability of such tissue, since following conventional corneal transplantation, the cornea-scleral rim containing PE and TZ are often discarded. Preclinical studies using appropriate animal models are required to examine the safety and efficacy of these tissue-engineered CEC-like grafts. This can be performed akin to the standard DSEAK and DMEK procedures using variously described carriers [25, 98, 99] or via simple cell injection [30, 100]. Before animal studies, the differentiated cells must be examined for CEC marker expression, since there is a substantial chance of cellular heterogeneity in non-optimal cultures. Stability of cell phenotype following passaging would also be important to assess, if one is considering the transportation of such cells [101].

However, there is no clear established animal model of corneal endothelial degeneration (e.g., FECD) that can correlate to the clinical situations in humans. Although the New Zealand albino rabbits share similar corneal parameters, such as CEC density and corneal dimensions, like that in humans, their corneal endothelia can regenerate after injury, hence inappropriate to be used for long-term efficacy study [25, 102]. Another option is the mini-pig, which also has anatomical ocular characteristics similar to humans and the CECs do not proliferate in vivo [103, 104]. However, intraoperative challenges related to high vitreous pressures, lack of corneal rigidity, and strong adhesion of DM to underlying stroma can significantly affect outcomes [105]. Non-human primates are the closest animal models to humans, regarding genetics, physiology, and behavior; however, the high cost and availability are the limitations for most research studies. Moreover, the treatment outcomes may be dependent on surgical manipulation, and, besides the recovery of endothelial functions, the testing of construct stability, cell purity, and viability at prolonged time intervals after transplantation are needed to be validated for its translation to clinical application.

## Conclusion

The functional corneal endothelium is essential to corneal transparency. Despite the advances of endothelial keratoplasty, the worldwide limitation of donor tissue supply is an urgent clinical unmet need for corneal endothelial failure and degeneration. The renewal of CECs from a source of stem cells or cells with stem-like characteristics is a promising therapeutic strategy to regenerate the functional corneal endothelium. However, more research into this field is required before it becomes a viable alternative to current definitive treatments. There is a currently insufficient understanding of the pathogenesis of endothelial degeneration diseases and whether they can be treated by targeted regenerative therapies. The molecular mechanisms that govern CEPs and their migration and differentiation to form mature CECs with efficient pumping activities are yet to be identified. Moreover, there remains a lack of identifiable markers for functional human CECs and their precursors. Methods of transplanting these cultured cells into in vivo models, also need to be studied further to determine their efficacy and viability following surgical manipulation, and whether they maintain the differentiated phenotype and cell function with long-term stability in vivo. This review has highlighted the latest discoveries and innovations in CEPs and bioengineering. The novel techniques presented here demonstrate the potential future treatments of CEC dysfunction.

## Data Availability

The data are available from the corresponding author upon reasonable request.

## Abbreviations

3D: Three-dimensional
CE: Central corneal endothelium
CEC: Corneal endothelial cell
CEP: Corneal endothelial progenitors
DM: Descemet’s membrane
DMEK: Descemet’s membrane endothelial keratoplasty
DMT: Descemet’s membrane transplantation
DSAEK: Descemet’s stripping automated endothelial keratoplasty
DSO: Descemet stripping only
EK: Endothelial keratoplasty
ESC: Embryonic stem cell
FECD: Fuchs endothelial corneal dystrophy
GMP: Good manufacturing practices
iPSCs: Induced pluripotent stem cells
Lgr5: Leucine-rich repeat-containing G protein-coupled receptor 5
NCC: Neural crest cell
OCT: Optical coherent tomography
p75^NTR^: p75 neurotrophin receptor
PE: Peripheral corneal endothelium
PK: Penetrating keratoplasty
POM: Periocular mesenchyme
POMP: Periocular mesenchymal precursors
ROCK: Rho-associated kinase
SEM: Scanning electron microscopy
SER: Selective endothelial removal
TEER: Trans-endothelial electric resistance
TGFβ: Transforming growth factor-β
TM: Trabecular meshwork
TZ: Transition zone
